# Multifaceted Modelling of Complex Business Enterprises

**DOI:** 10.1371/journal.pone.0134052

**Published:** 2015-08-06

**Authors:** Subrata Chakraborty, Kerrie Mengersen, Colin Fidge, Lin Ma, David Lassen

**Affiliations:** 1 School of Management and Enterprise, University of Southern Queensland, Springfield Central, Queensland, Australia; 2 School of Mathematical Sciences, Queensland University of Technology, Brisbane, Queensland, Australia; 3 School of Electrical Engineering and Computer Science, Queensland University of Technology, Brisbane, Queensland, Australia; 4 School of Chemistry, Physics and Mechanical Engineering, Queensland University of Technology, Brisbane, Queensland, Australia; 5 Queensland Rail, Brisbane, Queensland, Australia; Nankai University, CHINA

## Abstract

We formalise and present a new generic multifaceted complex system approach for modelling complex business enterprises. Our method has a strong focus on integrating the various data types available in an enterprise which represent the diverse perspectives of various stakeholders. We explain the challenges faced and define a novel approach to converting diverse data types into usable Bayesian probability forms. The data types that can be integrated include historic data, survey data, and management planning data, expert knowledge and incomplete data. The structural complexities of the complex system modelling process, based on various decision contexts, are also explained along with a solution. This new application of complex system models as a management tool for decision making is demonstrated using a railway transport case study. The case study demonstrates how the new approach can be utilised to develop a customised decision support model for a specific enterprise. Various decision scenarios are also provided to illustrate the versatility of the decision model at different phases of enterprise operations such as planning and control.

## Introduction

A complex system can be defined as a set of interconnected subsystems with dynamic interactions and a common purpose. The emergent behaviour of a complex system is dictated by various underlying factors. Complex system behaviours and features are well explained by Kauffman [1]. Complex systems sometimes can also be represented by graphs and the complexity measure can be achieved by graph entropies [2–5] based on Shannon’s Entropy theory [6]. Complex systems can be considered as multifaceted due to their prism-like ability to present certain aspects relevant to particular stakeholders, while preserving less significant features in the background.

**Fig 1:**
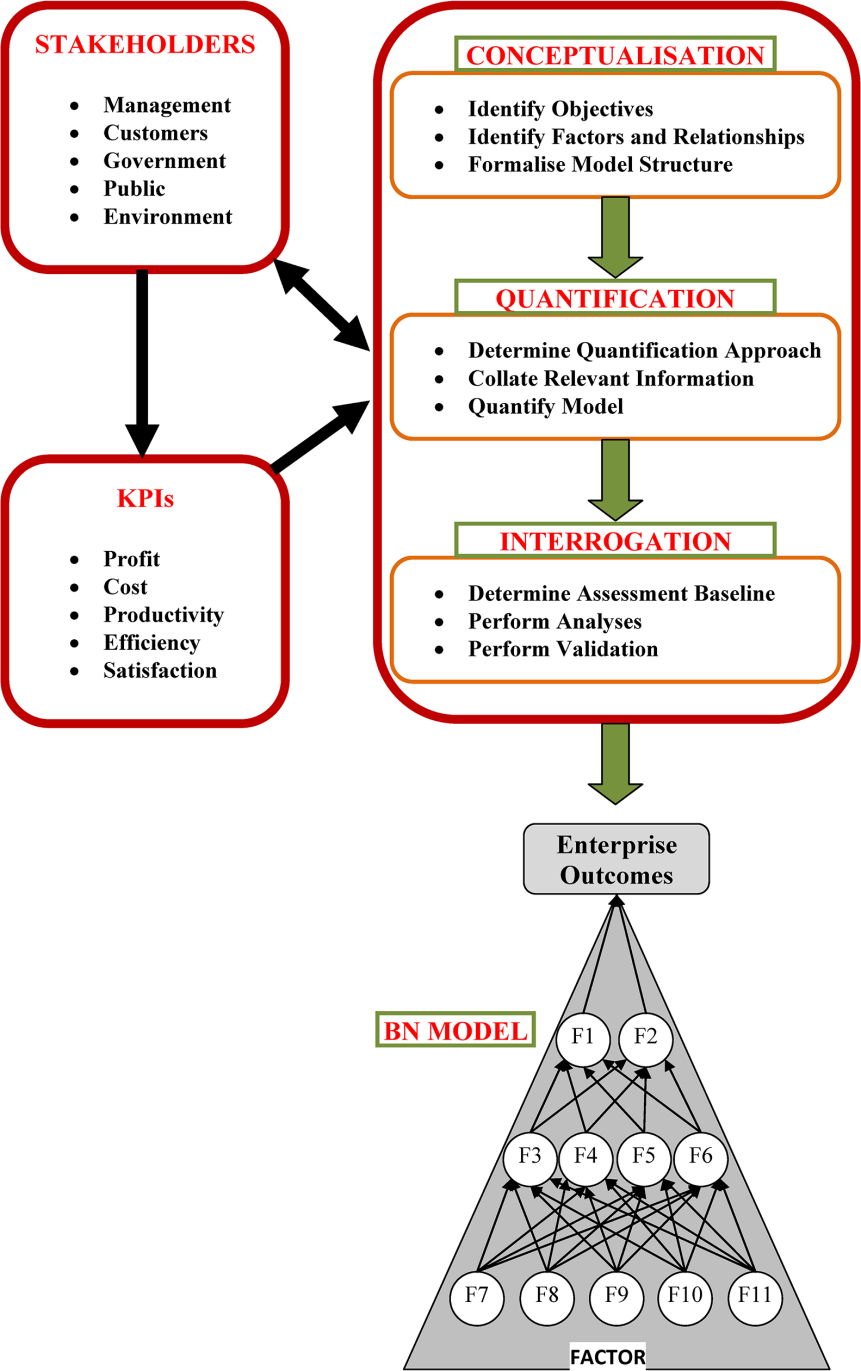
Complex System development approach for Business Enterprises.

A complex business enterprise can be considered similar to other kinds of complex system such as machines, ecology, the human body, etc. As shown on the left of Fig 1, a business system often interacts with multiple stakeholders such as management, customers, regulatory bodies, etc. Different stakeholders have different concerns, perspectives and expectations relevant to the business operation and performance. For example, management may be interested in better profit performance but the government may be interested in the system’s conformance to mandated regulations. The various perspectives of the different stakeholders often define the Key Performance Indicators (KPIs) of a business. KPIs such as profit, customer satisfaction, efficiency, etc. are measurable business performance indicators. There are various internal and external factors affecting the overall business goals. These factors are often organised in a multilevel structure with multidimensional interactions between them.

The multifaceted nature of business systems highlights the necessity of extensive analysis to understand various stakeholders’ perspectives, KPIs, factors affecting these KPIs, and the relationships between them in a business enterprise. Complex system modelling approaches have the capacity to accurately represent the multifaceted nature of a complex enterprise. Such models can provide a dynamic view of a complex business enterprise and highlight information appropriate to particular stakeholders. In such an enterprise it is understandably easy for stakeholders to lose sight of the overall objectives and interests of other stakeholders, and the implications of their actions on other processes and outcomes in the system. Complex system models can overcome this problem by providing an interactive, dynamic view of the whole system in order to bridge the gap between different stakeholders. In practice, complex system models can help decision makers to (i) identify various decision scenarios, (ii) assess plans, and (iii) assess performance.

Modelling complex systems is a challenging task. Key challenges include deciding the objectives, identifying various influencing factors, identifying relationships between factors, analysing available data, quantifying the model, developing the complex model, and communicating information and feedback. In this article we present a novel, generic complex system modelling approach which serves as a guideline for practitioners to develop specific models according to their requirements. Our approach is comprised of Conceptualisation, Quantification and Interrogation stages as shown in Fig 1. It is based on a standard Bayesian Network modelling technique but with a particular focus on integrating multiple data types representing the specific interests and concerns of different stakeholders. The resultant “multifaceted” models allow stakeholders to see not only the factors of interest to them, but also to see how their segment of the business is affected by, and affects, the rest of the enterprise.

In the remainder of this article we discuss related research in this area, present our new model development approach, and describe its application to a real-life case study from the transport sector.

## Related Work

Our work concerns modelling of business processes. In this section we review some of the large number of related approaches for assessing business performance. For example, Cost Benefit Analysis (CBA) has been used widely to assess projects for decision making in a wide range of business and non-business sectors. Early applications of the CBA method were presented by Prest and Turvey [7]. Recent applications of CBA include economic analysis of IT systems [8], the transport sector [9, 10], medical fields [11, 12] and ecological systems [13, 14] among others. The CBA method aims to assess decision scenarios and quantify monetary gains against costs for each scenario [15]. Although CBA was highly successful in these application domains, it has been criticised for its limited ability to handle complex situations with multiple stakeholders [16]. The monetary conversion process in CBA often fails to distinguish between direct and indirect impacts by treating them equally and it provides the decision maker with a false sense of certainty [16].

Considering the problems with the CBA method for benefits that are not directly financial in nature (such as social and environmental benefits), the Decision Rule method has been suggested as an alternative [17]. The Decision Rule method works by applying a set of rules to evaluate alternatives and find the best one [18]. However, the Non-Compensatory Decision Rule approach suffers from disadvantages including (a) limited applicability and (b) missing important information. The compensatory approach has been criticised because (a) overly complex judgements are required for rule development, (b) overview arguments may be difficult to define based on rules, (c) overall preference measures may be too abstract, and (d) the trade-off principle may not be well accepted by decision makers [19].

Business Process Modelling (BPM) has been widely used to model business systems [20, 21]. In the BPM approach, a business enterprise is viewed as a set of interacting processes representing various functions where the processes may be further decomposed [22]. An early BPM framework was proposed by Curtis et. al [23] in which processes are categorised as functional, behavioural, organizational and informational. Two distinct varieties of BPM approaches (i) static BPM [24] and (ii) dynamic BPM [25] are commonly used. Static modelling has been criticised firstly for assuming that business processes can only be designed in rational and technical terms, i.e., it neglects human and organizational issues; and secondly business processes are assumed to be static, i.e., models are simplified representations of business processes at a particular point in time [22]. Dynamic BPM overcomes some of these issues by introducing concepts such as interdependent, interactive, boundary-crossing, and super-ordinate goals in the modelling process [25]. BPM has been used for complex system modelling in diverse areas including education [26], manufacturing [27], information technology [28] and business management [29] among others. Simulation based Business Process Modelling approaches are also used to model complex systems [30]. Dynamic BPM suffers from a few disadvantages for modelling real world complex systems: (i) it may lead to the neglect of the socio-political dimension of a business process, as there is an implied belief that a business process can only be approached in logical and rational terms; (ii) such approaches obviously have a cost, so the time and skills required to build a dynamic computer model of simple systems may not add any value over simple flowcharts or spreadsheets; and (iii) it ignores the feedback loops that may determine the behaviour of many real-world business processes [22].

Multi Criteria Decision Making (MCDM) methods have been applied in many practical decision making situations, by practitioners and academics [31], including portfolio management [32], energy management [33], ecology [34], etc. MCDM methods generally compare several decision options against multiple and often conflicting criteria to provide decision outcomes in terms of ranks or overall scores. Complex systems are also modelled using MCDM approaches including object-oriented modelling [35], modelling socio-economic processes [36], modelling production-inventory-supply chain systems [37], maintenance process modelling [38], etc. MCDM methods have the ability to incorporate the performances of decision alternatives under various criteria in easy-to-use processes for finding the best decision. MCDM methods may be able to quantify system performances from diverse measurements of subsystem performances but are unable to maintain the interrelationships for decision purposes as the overall decision outcome is obtained by combining the available information and the individual information is lost in this process [31].

The multi-agent technique is a relatively new complex system modelling paradigm that utilises the autonomy and characteristics of various entities in a system along with their relationships with each other [39]. The multi-agent technique has been widely used in supply chain management [40, 41], manufacturing [42–44], and environment and ecology [45, 46]. Advantages of multi-agent modelling include the ability to model a system in a realistic form, inclusion of heterogeneity while incorporating behaviours of different agents, flexibility and scalability of the developed models, and the ability to incorporate local objectives within the systems [39]. On the other hand some known disadvantages include extensive data requirements [47] and effort required for modelling, and resulting models which are developed for specific contexts and have very limited generic usage [48].

The various decision support and complex system modelling approaches discussed above each have distinct advantages and disadvantages and have been used in diverse application domains. These approaches have been used to develop models with specific stakeholder requirements in mind but they have limitations in some aspects of flexibility in usage and the extensibility necessary for developing multifaceted models. In a multifaceted modelling approach the same model incorporates information related to all the stakeholders and can highlight and show those factors appropriate for a particular stakeholder while also revealing the interconnections and decision impacts on other stakeholders. For example an organisation may have multiple units (operations, finance, customer service, etc.). If a complex system model for the organisation is developed by following the organisational structure it will contain operational, performance and resource information for each department. To maximise the value of the modelling effort, the resulting model should also be sufficiently flexible that it can be used for purposes not anticipated at the time of its construction, and it must be maintainable as the business grows and evolves.

In our approach we use Bayesian Network modelling. A Bayesian Network modelling formalism has the inherent ability to model multifaceted complex systems by virtue of its simplicity and generality. They are used in various research and practical areas [49–52]. The advantages of this approach include the ability to model complex interrelations between factors and the sensitivity of factors on decision outcomes [53], the ability to perform scenario analyses, to undertake sophisticated interrogations of the system, and to include other sources of information in the model, such as observational and experimental data, results from previous experiments, knowledge from published literature, expert judgements, and so on.

However, such a formal modelling notation alone is not useful without a well-defined strategy for constructing, employing and maintaining models based on the data available from the business enterprise. In the following section we present a novel, multifaceted approach for modelling complex business enterprises based on a Bayesian Network notation.

## Model Development Approach

Here we describe our development approach for constructing multifaceted complex system models from diverse available data in three stages: (i) Conceptualisation, (ii) Quantification, and (iii) Interrogation. The Conceptualisation stage shows how each small facet of a system is built based on collected information and how small model structures are joined together to build the large model of the whole system. In the Quantification stage diverse data related to various facets of the system are identified, processed and incorporated into the model in probabilistic forms. The Interrogation stage involves extracting business insights from the model to help a decision maker to understand how his decision impacts on his area (one facet) as well as impacts on others in the organisation. This understanding improves the cohesion within an organisation and reduces decision conflicts. The model stages are described below and are shown in Fig 1.

### Stage 1: Conceptualisation

In this first stage of our development approach the model of the complex system’s structure is developed as a set of interconnected nodes where the connections represent causal influences. The model is constructed through the following three steps.

#### Identify objectives

The top level nodes of the model are identified as the objectives of the complex system. These objectives are typically related to the business’s KPIs, thus representing the vested interests of different stakeholders. Often system modellers tend to merge top-level objectives into a single overall system objective node which helps to highlight the whole system’s dynamics.

#### Identify factors and relationships

In this step the key factors influencing the system objectives are identified. Often a single factor may influence multiple objectives and an objective may be influenced by multiple factors. The higher level factors may in turn have multiple lower level factors influencing them. The complete set of factors in a complex system is identified and the relationships are defined as their influencing pattern.

#### Formalise model structure

The model structure depends on how the factors are organized in the model. The model structure is identified based on the decision perspectives. Sometimes the same factors can appear in different structures based on the decision analysis requirements. In some cases the model structure can be learnt from the data [54] but more often the appropriate model structure is determined by, or at least requires validation from, the decision maker (domain expert).

### Stage 2: Quantification

In this second stage of our overall development approach the available data is processed into measurable forms and linked to the relationship model developed earlier in order to support quantifiable analyses and assessments of the model. This is done in the following three steps.

#### Determine quantification approach

In this step an appropriate information quantification approach is selected. Some commonly applied approaches include summary statistical evaluations, factor analysis and its variants including customer satisfaction indices [55–57], linear regression and its variants [58, 59], non-parametric non-linear approaches such as classification and regression trees [60–62], latent factor approaches such as structural equation models [63], multicriteria approaches [64], and so on. While these approaches offer many insights into the system of interest, they do not focus on modelling the system as a whole [65, 66]. In many ways, a Bayesian Network borrows from all of these approaches to create a more flexible modelling environment and a whole system approach.

**Fig 2:**
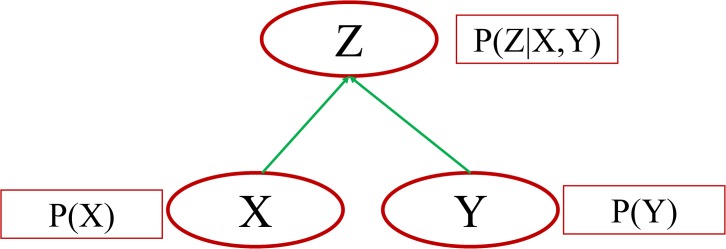
Example of a Bayesian Network (directed acyclic graph).

Bayesian Networks are also known as recursive graphical models, belief networks, causal probabilistic networks, causal networks and influence diagrams among others [67]. A BN can be expressed as two components, the first qualitative and the second quantitative [68, 69]. The qualitative expression is depicted as a directed acyclic graph (DAG), which consists of a set of variables (denoted by nodes) and relationships between the variables (denoted by arcs) [54]. The quantitative expression comprises probabilities of the variables. Fig 2 shows a Bayesian Network with three variables *X*, *Y* and *Z*. Variables *X* and *Y* are parents for variable *Z*, which indicates that *Z* is the dependent node. The probability for *Z* is a conditional one based on the probabilities of *X* and *Y*.

The probabilities in a Bayesian Network are simplified by the DAG structure of the BN, by applying directional separation (d-separation) [70] and a Markov property assumption [71, 72], so that the probability distribution of any variable is solely dependent on its parents. Thus, the probability distribution in a BN with *n* nodes (*X*_1_, …, *X*_*n*_) can be formulated as

$$P(X_{1},\;\ldots \;,X_{n}=\prod_{i=1}^{n}P(X_{i}|P_{a}\left(X_{i}\right))$$

where *P*_*a*_ (*X*_*i*_) is the set of the probability distributions corresponding to the parents of node *X*_*i*_ [72, 73].

For Fig 2 the above equation can be written as

P(Z)=P(Z|X,Y)*P(X)*P(Y).

#### Collate relevant information

Information about complex systems is often available from different sources and in different forms including historic records, survey results, operational plans and expert knowledge. In some situations appropriate data is not available in a usable form, which we classify as incomplete or missing data. These multiple data types often represent the separate perspectives of different stakeholders in a shared enterprise. Some key information types are described below.

Historic data are extant records of past incidents. With thorough investigation into historic data we can sometimes identify causes and effects of an incident and use this knowledge to help construct the model. We can also identify the consequential severity of various causes which can help with the model’s quantification.

Survey data is used to identify five key features of survey questionnaires which can guide model development. First, the questionnaire itself can provide a clear description of the objective of the survey. Second, the questions themselves can assist in the definition of key explicit factors. Third, the grouping or categorisation of questions can assist in the identification of key latent factors. Fourth, a well-structured questionnaire can provide guidance about the relationships between these factors. Finally, an attitudinal questionnaire typically facilitates quantification of the corresponding model.

For example, responses may be categorical (e.g., gender) and hence the proportions of responses in each category can be used as probabilistic estimates in the Bayesian Network. Alternatively, responses with an underlying continuous scale (e.g., age) may be treated as continuous or discretised into a set of ordered categories, such as Young and Old, where these are appropriately defined. Attitudinal responses measured on a Likert scale can be similarly assumed to be approximately continuous, depending on the context and scale, or discrete with the number of categories equalling the number of levels of the scale or a smaller number of categories representing, for example, High, Medium and Low, or Good and Bad. Finally, qualitative responses can be generally sorted into categories and included in the model.

Planning data reflects the future state of a system. For instance, a railway’s operational planning data provides the capacity and utilisation of rail networks. Maintenance plans provide indications of planned delays. With proper utilisation of planning data we can identify the stress points in a complex system thus helping the management plan accordingly.

Expert knowledge is vital in developing complex system models. Context specific behaviours of any dynamic system are often captured through expert involvement. Often other data forms cannot express a system’s characteristics completely and experts with working knowledge are invaluable in filling the gaps. Expert knowledge is highly applied in validation of BN models.

Missing data in BN modelling can be classified as data that is required but not available due to the absence of past records, or data that is not accessible due to data protection regulations or because it has become corrupted over time. It is important to identify missing data for any BN model as it provides the decision maker with information regarding the limitations of the developed model and shows the completeness of it. Often BN models can be used to understand various decision scenarios with simulated or estimated values for missing data.

#### Quantify model

This step involves Bayesian Network model quantification, including defining and estimating the probability distributions for each factor. Continuous distributions can be represented in full, or can be discretised into levels or categories that are meaningful in a business context. This allows both continuous and discrete distributions to be treated in the same manner in the network, in that they can be represented as marginal probability tables for the nodes with no parents (terminal nodes), and conditional probability tables (CPTs) for the nodes with parents.

The CPTs can be estimated in a number of ways, using direct or indirect methods. We describe a generic BN quantification approach developed for data-type specific quantification approaches.

Historic data quantification identifies nodes (factors) with historic data using the model structure. The level for these nodes and their possible probability states (such as High, Medium, and Low) are identified. For the lowest level nodes state probabilities are calculated as proportions of the total records available. The intermediate node states are estimated based on defined weighted relationships. Indirect approaches are applied for missing information [74].

Survey data quantification defines the levels for nodes with survey data in the model structure and their possible probability states. For the lowest level nodes state probabilities are calculated as proportions of total survey respondents. The intermediate node states are estimated based on defined weighted relationships. Indirect approaches are applied for missing information.

Planning data quantification identifies the levels for nodes with planning data in the model structure and their possible probability states. For the lowest level nodes state probabilities are calculated using direct approach. The intermediate node states are estimated based on defined weighted relationships. Indirect approaches are applied for missing information.

Expert knowledge quantification identifies the levels for nodes requiring expert knowledge and their possible probability states. For the lowest level nodes state probabilities are calculated through a standard expert elicitation approach such as that described by [75]. The elicited knowledge is then added into the BN model and verified with multiple experts where possible.

Missing data quantification identifies the levels for nodes (factors) without available data and their possible probability states are estimated based on expert inputs. Initial values are set as equal for all the states and exploratory simulations are conducted by controlled variations of the states’ values. Based on expert opinion and feedback about the resulting model’s behaviour, acceptable value ranges for specific decision context are then defined.

### Stage 3: Interrogation

The third stage of our overall development approach consists of using a well-structured and quantified complex system model. It comprises the following three steps.

#### Determine assessment baseline

The assessment baseline refers to the initial state of the model. Once the model’s structure is formalised and quantified, the Conditional Probability Tables for each node are considered to be the assessment baseline. Any analysis result is compared with this baseline to understand the changes in the results.

#### Perform analyses

A range of analyses can be performed with a well-structured BN complex system model [54, 76–78]. These analyses help the decision maker assess future plans and impacts as well as undertake performance assessments of past decisions. Some key analyses are described below.

Sensitivity analysis is used to understand responsiveness of the model. It helps the decision maker by identifying the most sensitive factors.

Influence analysis helps to understand the magnitudes of impacts of parent nodes on their respective child nodes and vice versa.

Scenario analysis, also known as what-if analysis, is used to assess impacts in the system caused by any change. The decision maker use this analysis to view potential impacts of his planning decisions. It is also used for performance assessments of past decisions.

#### Perform validation

Validation of the model is essential to ensure that the results it produces are meaningful [48, 68]. Validation is performed in two steps (i) Model Validation, and (ii) Results validation.

Having understood the model’s dynamics, the next step is to independently validate its correctness within the application domain. The validation process includes: (i) Validation of the objective which is often based on business objectives defined by domain experts, (ii) Validation of the structure which is often done by domain experts, and (iii) Validation of quantified data which is done typically based on expert judgement and past consolidated reports.

Validation of analysis results is often performed through assessments of the outcomes by domain experts. Past results are sometimes compared with those from the model to assess the validity of the analysis results. (One strategy when developing a model from historic data is to divide the historic log in half, use the first part to construct and quantify the model, and then assess how well the model can predict the behaviours found in the second half of the log.)

An important step of the complex system modelling process is to create the modes by which the BN results will be communicated to business managers and other stakeholders. Three such approaches are suggested here based on our experiences. First, the model itself can be used as an interactive software tool. There are now many software packages for developing a BN in this manner, including GeNIe [79], BayesiaLab [80], Netica [81] and Hugin [82]. Second, general information templates can be constructed and then tailored for specific situations. These templates may be in the form of a report containing the BN model, the conditional probability tables, the analytic results, and the validations. Alternatively it may represent a more concise summary of the outcomes; one option is to create a form of “management dashboard” in which the performances of the key nodes can be depicted numerically and/or visually. For example, a traffic light colour coding system could be used, with red, orange and green indicating respectively poor, moderate and good performance.

## Case Study

Having presented our model development approach, in this section we illustrate its practical application to a real-life case study from the transport sector. Railways are one of the major modes of mass transport worldwide. “On time service” is vital to any railway transport system. Any delay in the service generally causes significant effects on the public and businesses. In order to minimize transport interruptions and maximize operational efficiency, it is necessary to obtain a detailed understanding of the causes and effects of rail delays. In this industrial case study we identified various perspectives and factors causing delays, the relationships between them, and their significance. The outcomes will provide railway management with the capacity to identify key decision areas for performance improvements.

A variety of data regarding the causes and effects of delays in railway transport are available from multiple sources and in multiple formats such as incident records, surveys, operational plans and expert evaluations. We applied our BN-based modelling approach to integrate these multiple data types into a single complex system model.

Queensland Rail is one of the major passenger railway providers in Australia with a large number of scheduled trains operating every year. Timely operation is a key performance indicator for the organization. In order to better understand the causes of operational delays and to improve performance, Queensland Rail engaged us to conduct a delay analysis study. The study focused on understanding the key factors behind delays and their relationships to the overall operations of Queensland Rail. In this context we note three recent incidents each of which highlights the interconnectedness of events in a complex rail system.

Incident 1: During evening peak hour a pigeon was electrocuted on an overhead power line in the Brisbane city center which brought down the whole city network for an hour.

Incident 2: A slight delay on the Gold Coast-Airport line due to bad weather resulted in a ripple effect which caused several airport-bound trains to be late and hundreds of passengers to miss their flights.

Incident 3: A foot bridge over a fenced rail line was closed suddenly due to maintenance work on the rail line, forcing residents to walk via a much longer alternative path. Unhappy residents took this seemingly minor issue up to the ministerial level, resulting in considerable political pressure on Queensland Rail.

These incidents highlight three major aspects of Queensland Rail operation. Incident 1 shows the significance of key physical locations in the rail network and the need for understanding them better. Incident 2 shows the significance of priority services and Incident 3 shows the interaction of Queensland Rail’s operation with the broader society, beyond its passengers.

To try to understand such relationships, we applied our modelling approach using data made available to us by Queensland Rail. In the following sections we describe the complex system model we developed and its application.

### Study Objective and Available Data

We developed a BN based complex system model for Queensland Rail’s enterprise-level Key Performance Indicators to understand how they are affected by any delay in the system. We used diverse data sources made available from Queensland Rail to construct the Bayesian Network. As per the *Conceptualisation* stage we defined the objective as “Impact of Delay”, which translates into the topmost node for our model. The data available for this model are described below, as per the *Quantification* stage.

#### Historic data

We obtained Queensland passenger rail delay records for a period of one year (2010). There are 29,735 records, one for each affected train. The key data components of each delay incident record include an (a) incident number, (b) incident date, (c) train directly involved in the incident, (d) other delayed train due to the incident, (e) incident category, (f) incident sub category, (g) amount of time lost, (h) time of the day, and (i) line affected.

We also obtained the passenger load survey data for 3 years (2008–2010). The 2008 data is in a yearly format but 2009 and 2010 data was in a quarterly format. This load data provides information about the number of passengers at each station and on various rail segments.

#### Survey data

Queensland Rail’s customer experience survey consists of a set of questionnaires. Passengers were asked about their experiences regarding various attributes (factors) of QR customer service. These factors are currently divided into two levels as shown in Table 1.

**Table 1:** Customer service attributes considered in the survey.

Attribute No.	Level 1 attributes	Level 2 attributes
1	Carriages	Cleanliness, Lack of wear and tear, Entertainment, Air-conditioning, Feeling safe and at ease.
2	Station—Central Business District (CBD)	Cleanliness, Ease of Access, Lighting, QR Staff, Greenery.
3	Access to CBD Station	
4	Platform—Suburban	Condition of surface, Shelter
5	Access to Suburban Station	
6	Station—Suburban	
7	Ticketing	
8	Platform—CBD	
9	Signage and Information	
10	Bus Connection	
11	Entry onto Train	
12	Pre-trip information	
13	Parking	
14	Disembarking and Exit	
15	Efficiency of service	
16	Services on time	
17	Reliability	
18	Frequency of service	
19	Safe operation	
20	Helpfulness of staff	
21	Respect for passengers	
22	Affordability	

The survey was conducted with a large number of passengers (1000+) travelling during peak and off-peak times. The off-peak time was defined as 9:00 am to 3:30 pm and after 7:00 pm until 2:00 am the following day on weekdays and all day on weekends and gazetted public holidays. All other times are considered as peak time. For each service factor, the survey respondents said whether they had had Positive or Negative experiences. The relative importance of each factor was provided by experts at Queensland Rail.

#### Planning data

Relevant (2011) passenger train load planning data was obtained for each rail line. This loading plan includes loads at various times of the day (AM, PM) and weekends. Relevant (2011) freight train load plans for each line were also obtained. The plans provide total loads for each line during weekdays and weekends.

#### Expert knowledge

The interrelationships between factors were identified through several workshops and interviews with domain experts at Queensland Rail. The significance of factors in terms of weights were also elicited through expert involvement.

#### Missing data

Usable safety incident data was not available to us, although this is obviously a very important consideration. Similarly, Level 2 attributes from Table 1 for customer survey data was not available for all the factors.

#### Model structure development

As per our *Conceptualisation* stage, we identified five key factors affecting the model objective as *Safety Incidents*, *Customer Service Impact*, *Social Impact*, *Cost of Disruption*, and *Operational Disruption*. The remaining sub-factors for each of the key factors were then identified. Relationships and node states for each node were then defined. The sub-factors, node states and relationships for *Cost of Disruption* and *Operational Disruption* were defined utilising historic and planning data along with expert knowledge. The *Customer Service Impact* node and its sub-factors and relationships were defined using survey data and expert knowledge. Expert approximations were applied for defining the characteristics of *Social Impact*. The *Safety Incidents* factor was identified as a key factor through discussions with domain experts but due to lack of available usable data, we decided to conduct a simulation study to understand its impacts.

#### Formalisation of model structure

**Fig 3:**
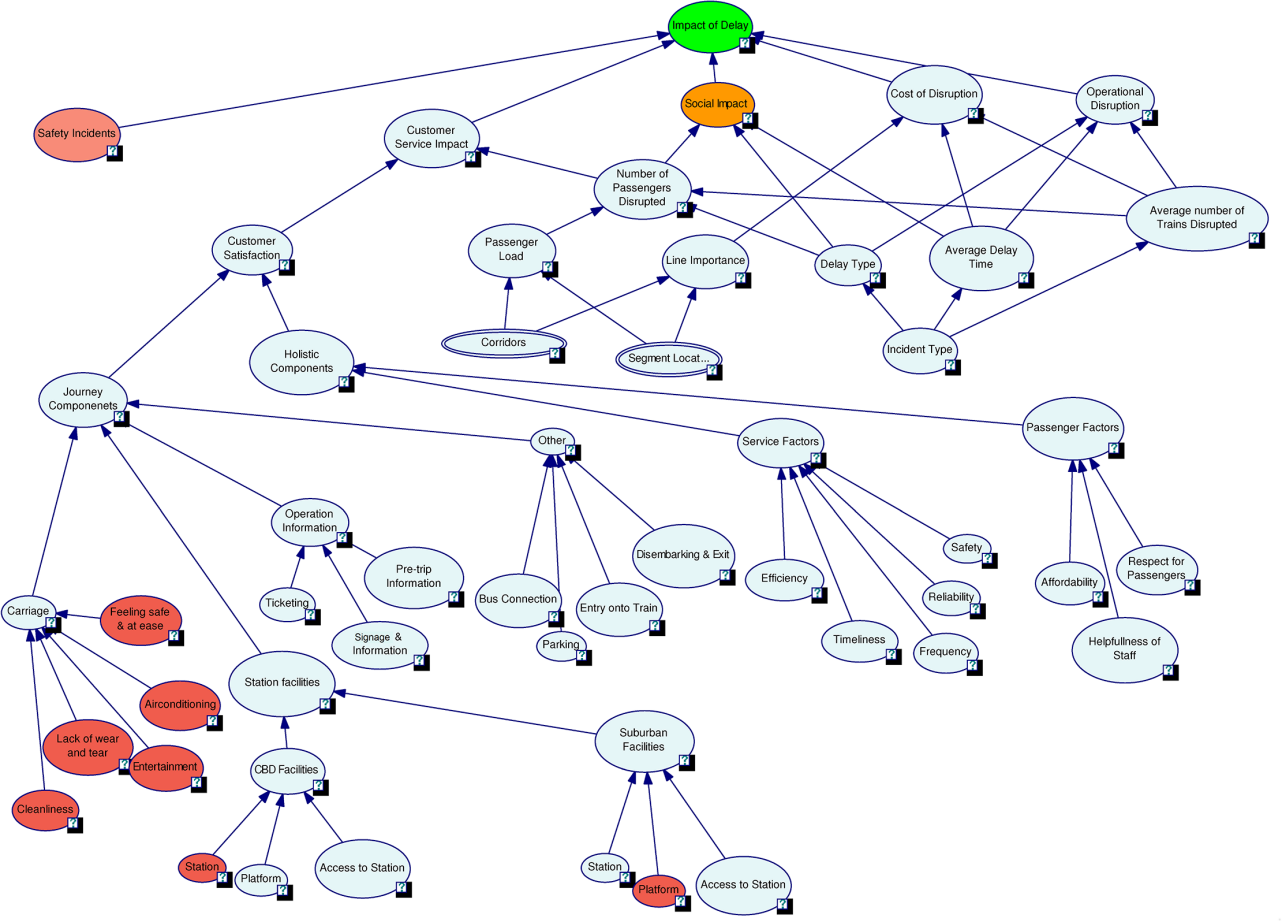
Complete delay impact model.

Based on the initial system model, a set of workshops were conducted with groups of experts from different operational areas within Queensland Rail. The model was tested and expert advice regarding nodes, relationships and node states were considered for model improvements. The final model shown in Fig 3 is the result of several such iterative workshops.

#### Model quantification

With the model’s structure in place, it was then necessary to quantify it, as per our *Quantification* stage.

With historic data quantification the Conditional Probability Table for parent node *Incident Type* was defined based on the historic data which had 22 known causes for an incident. The probability of each incident type was calculated as the proportion of a particular incident type within the whole data set. For example, the total number of incidents is 11799 and the total number of incidents due to incident type “Emergency Services” was 1003 so the probability of an “Emergency Services” incident to occur was 1003/11799 = 0.085007.

The CPTs for intermediate nodes such as Delay Type, Average Delay Time, etc. were then quantified using cross classification. Ranges for node states were often defined using a certain percentile of the recorded data. Appropriate weights obtained from experts were also incorporated into the conditional probability tables.

With survey data quantification using available customer service data we constructed the CPT for each node and estimated the probabilities. For the parent nodes, the probability tables were quantified using the proportion of customers with Positive, Neutral and Negative satisfaction responses in the survey data. Weights for these nodes were determined as 0.52, 0.16 and 0.32; these were provided by Queensland Rail experts based on past analytical knowledge. These weights were used to approximate the CPTs for the intermediate nodes.

With planning data quantification we defined parent nodes Corridors and Segment Location as deterministic nodes due to the nature of available data. The states of these nodes and their child nodes were defined using classification of the planning data available.

With expert knowledge quantification the Social Impact node is dependent on factors Delay Type, Number of Passengers Disrupted and Average Delay Time. Social Impact was defined as Critical, Moderate and Minor. The probabilities of the influencing factors were weighted as Delay Type = 0.4, Average Delay Time = 0.3 and Average Delay Time = 0.3 following expert advice. The probabilities of Critical, Moderate and Minor for Social Impact were calculated based on the probabilities of the three influencing factors using defined rules. For example, if Delay Type is “Peak”, Average Delay Time is “Low” and Average Delay Time is “High” then the probabilities for a Social Impact incident will be Critical = 0.7 and Minor = 0.3.

With missing data quantification safety data was not available for the development of this model but it was identified as a key factor affecting delay impacts. In order to keep the impacts of the node *Safety Incidents* neutral, the states critical, moderate and minor were given probabilities 0.33, 0.34 and 0.33. Simulations were conducted by changing the values of each state of the Safety Incidents node from 0 to 100 in steps of 10 and the changes in the states of the topmost node, *Impact of Delay*, were noted. The results indicated linear relationships between the states of these nodes. The five Level 2 nodes (Safety Incidents, Customer Service Impact, Social Impact, Cost of Disruption and Operational Disruption) impacting the topmost node Impact of Delay were given equal weights. Simulations were also conducted by varying the weights of the Level 2 nodes and the results showed a linear relationship between them and the top node. The simulation results affirm the linear model structure between these nodes as per Fig 3.

For the node *Carriage* in Fig 3 the overall probability values for its three states were available from survey data. There are five parent nodes for Carriage namely Cleanliness, Lack of Wear and tear, Feeling Safe and at ease, Entertainment and Air-conditioning. There was not enough available data to estimate the probability values for states of these parent nodes so they were considered to have the same values as the child node Carriage. Simulations were conducted by systematically varying the states’ probabilities and relative weight. The simulation results confirm linearity between Carriage and its parent nodes.

**Fig 4:**
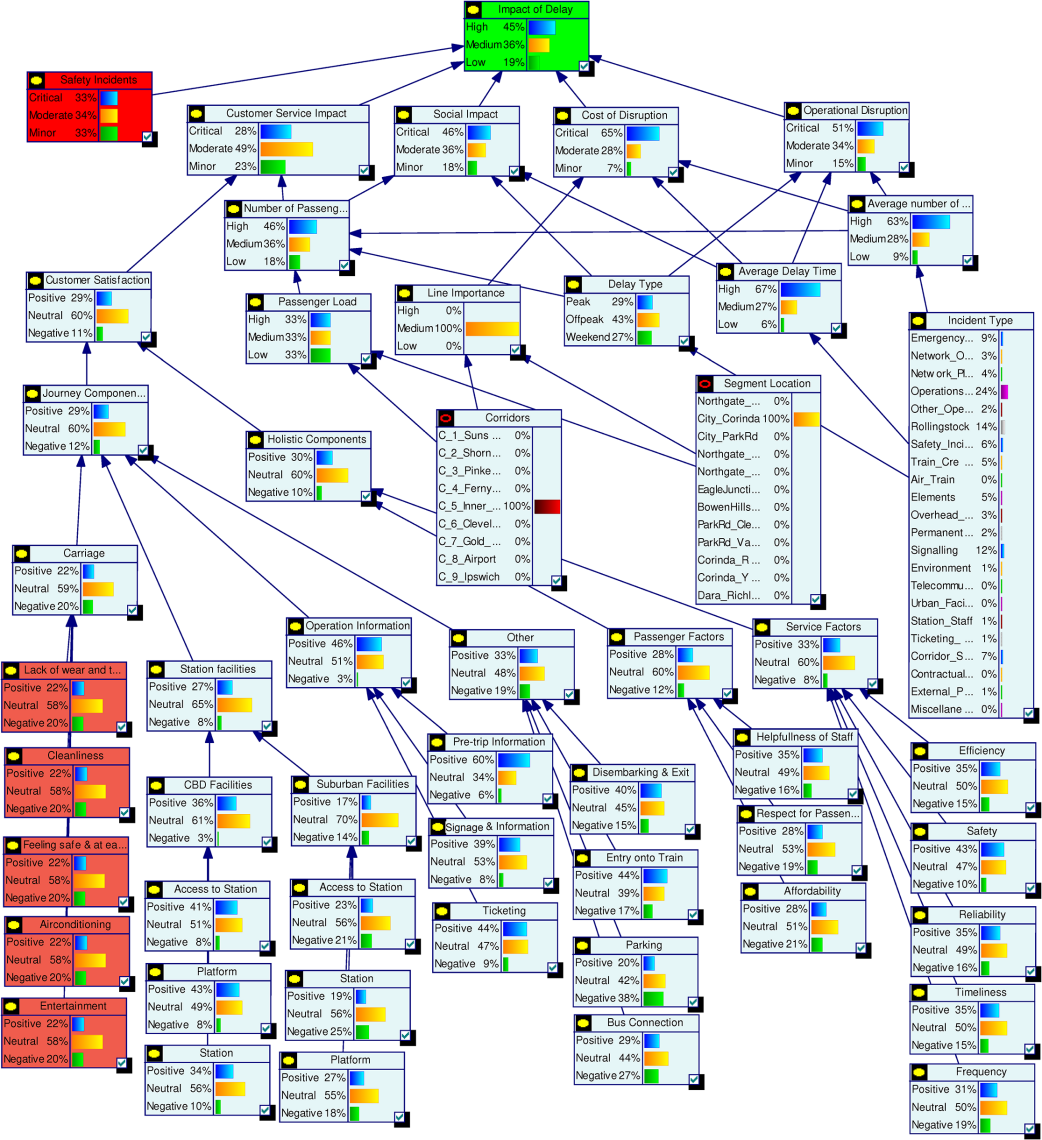
Complete model with quantified data.

Impact of Delay is the final node which represents the overall probabilistic effects of all factors. It is directly dependent on five key factors Operational Disruption, Cost of Disruption, Social Impact, Customer Service Interruption and Safety Incident. The Impact of Delay is defined as High, Medium and Low. The five influencing factors are equally weighted and each given a probability of 0.2. The probabilities of High, Medium and Low for Impact of Delay are calculated using defined rules. For example, if Operational Disruption is “Critical”, Cost of Disruption is “Moderate”, Social Impact is “Minor”, Customer Service Interruption is “Critical” and Safety Incident is “Minor” then the probabilities of Impact of Delay will be High = 0.4, Medium = 0.2 and Low = 0.4. Fig 4 shows the probabilities estimated for the complete model.

#### Model interrogation

The Bayesian Network model developed in the previous section can be used as a management tool for decision support as per our *Interrogation* stage. Various analytical capabilities of this model are demonstrated in the following sections.

**Fig 5:**
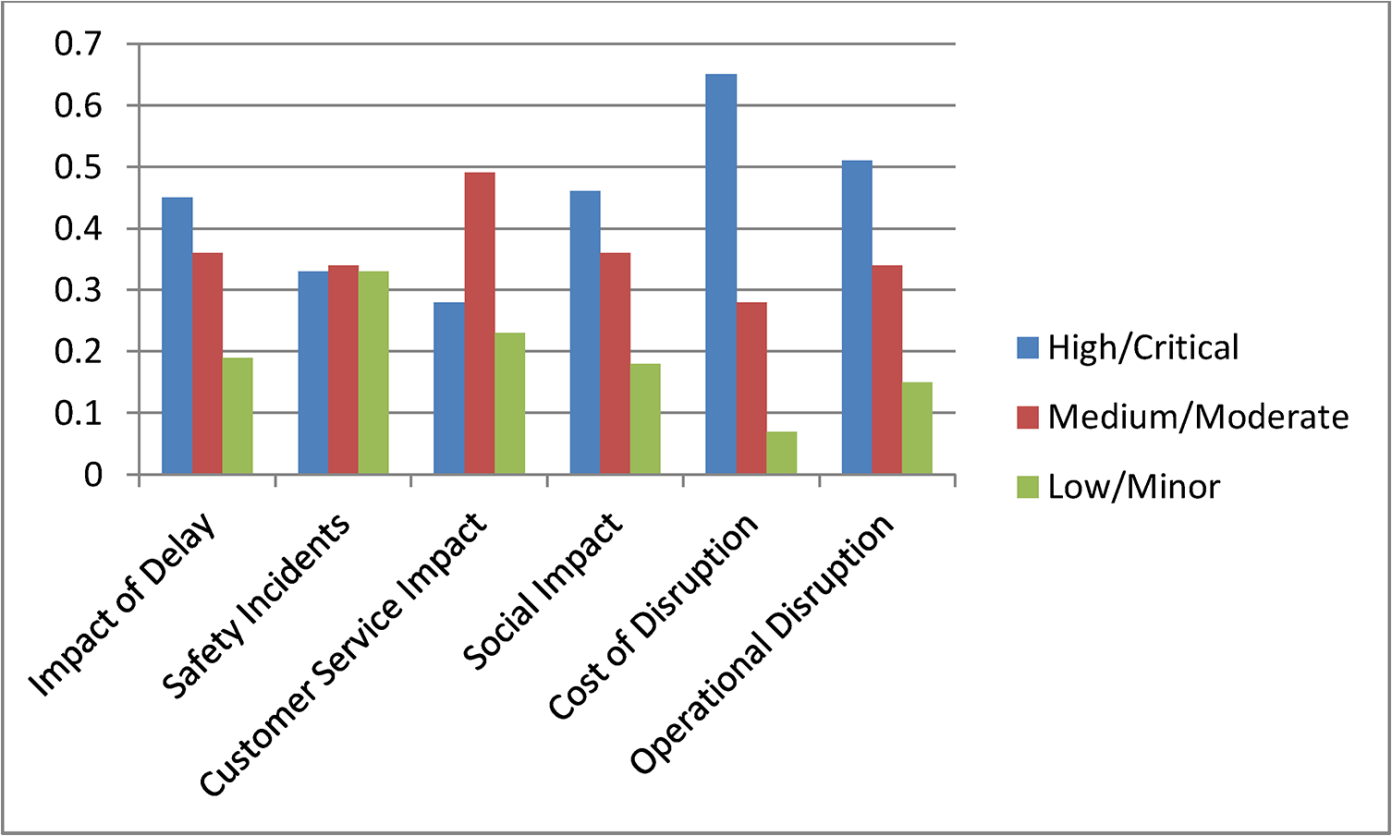
Initial probability values for top level nodes.

Current status assessment shown in Fig 4 depicts the initial probability settings for the complete model. In Fig 5 we summarise the state probabilities of the topmost node Impact of Delay and the five Level 2 nodes Safety Incidents, Customer Service Impact, Social Impact, Cost of Disruption, and Operational Disruption. This table is used as a base state to understand the changes during various scenario analyses. In the base model we have included higher probability values for states being High or Critical for the nodes Impact of Delay, Social Impact, Cost of Disruption and Operational Disruption.

**Fig 6:**
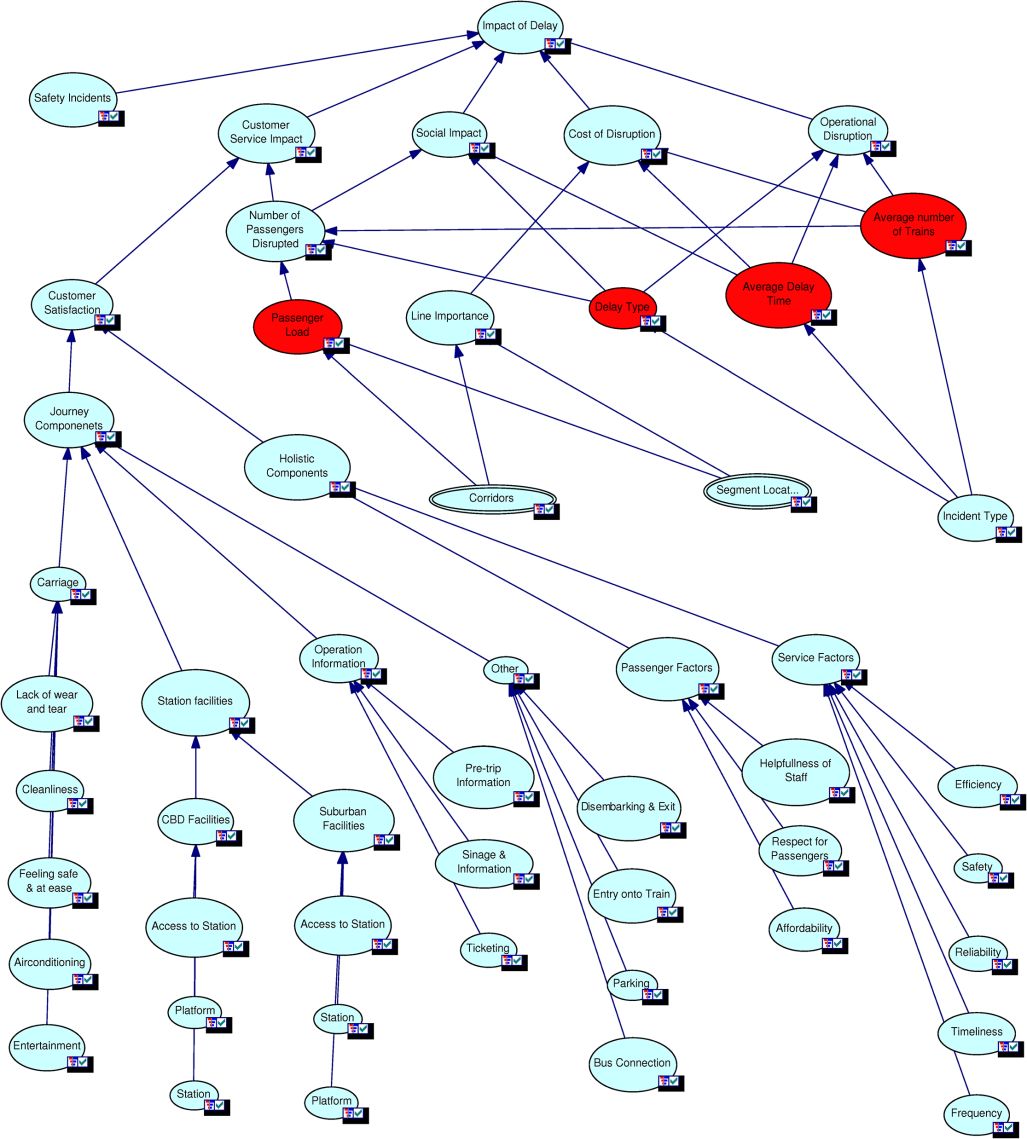
Sensitivity analysis on the base model.

A sensitivity analysis test was conducted on the base model configuration, as shown in Fig 6 where the most sensitive nodes are shown in red. The nodes Passenger Load, Delay Type, Average Delay Time, and Average Number of Trains were found to be the most sensitive. This analysis helps the decision maker identify nodes that are sensitive to changes, thus extra care can be taken when estimating their probability values. Similar sensitivity assessments can be conducted on the model under changed circumstances to understand the sensitivity of the model.

**Fig 7:**
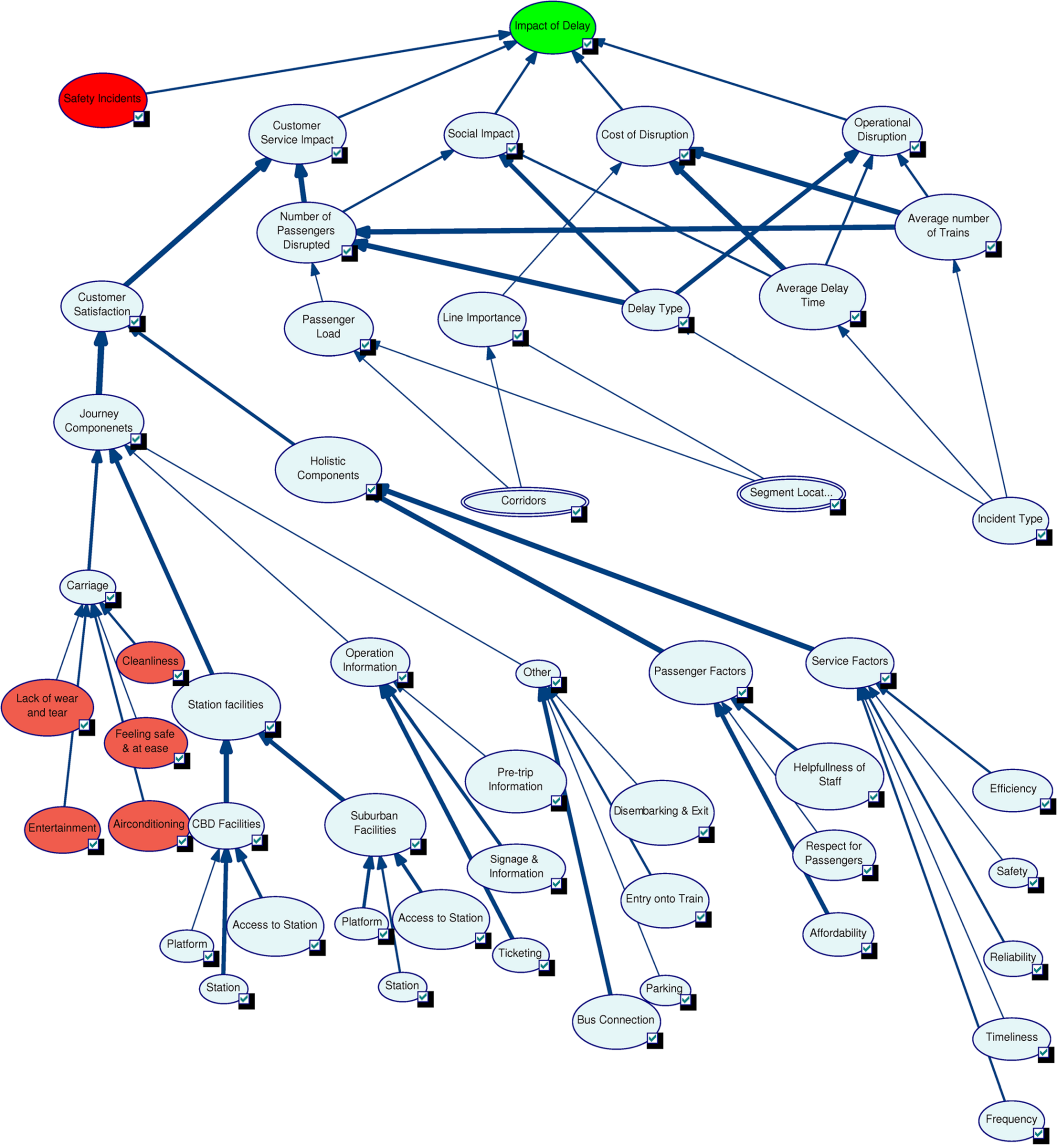
Influence analysis on the base model.

An influence analysis on the base model identified the level of influence of each node on its child nodes, as shown in Fig 7 where line thickness corresponds to degree of influence. The results can be summarised as follows.

1. The top most node *Impact of Delay* has equal influences on it from its five parent nodes.
2. The Customer Service Impact node is heavily influenced by the Number of Passengers Disrupted and Customer Satisfaction nodes. The Customer Satisfaction node is influenced most by the Journey Components node which in turn is highly influenced by the node Station Facilities. The Station node has a strong influence on CBD (Central Business District) Facilities. The nodes Ticketing, Bus Connection and Affordability have the strongest influences on Operation Information, Other, and Passenger Factors respectively.
3. The *Social Impact* node has its strongest influence from the *Delay Type* node.
4. The *Cost of Disruption* node is highly influenced by the *Average Delay Time* and *Average Number of Trains* nodes.
5. The *Operational Disruption* node is highly influenced by the node *Delay Type*.

Scenario analyses were conducted on a set of realistic scenarios were created from expert interviews with Queensland Rail. These scenarios were analysed in the model by setting the necessary scenario parameters and recording the results. Five such scenarios are presented below.

**Fig 8:**
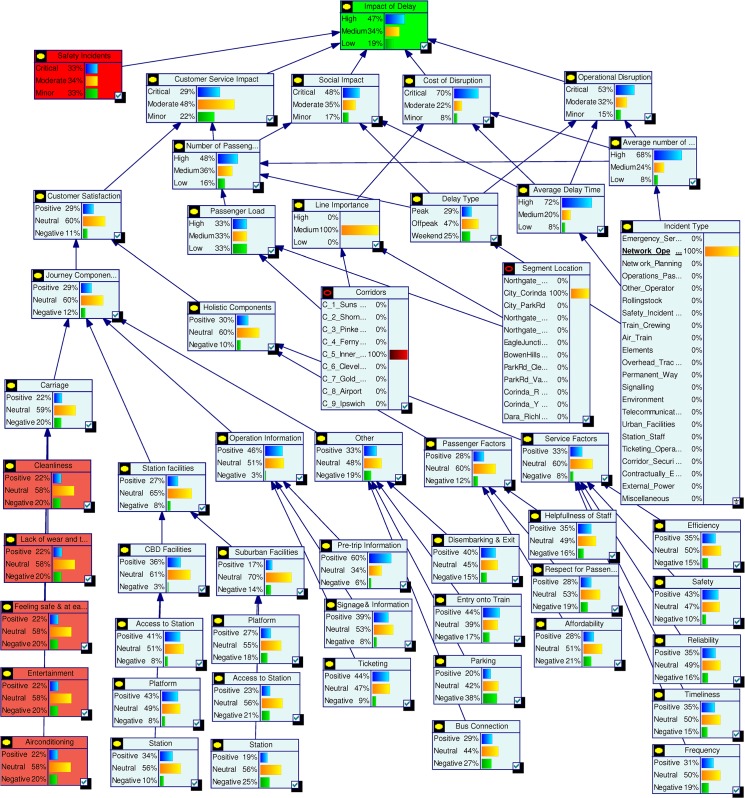
Analysis results for Scenario 1.

Scenario 1: **(**Assessing a known incident) Assume that there has been an incident on the railway network and the management received confirmation that the incident was due to problem with network operations. With this confirmed information we interrogated the model by setting the evidence in the node Incident Type as Network Operation = 100%. Comparing the results with the base model probabilities in Fig 5 we observe that the new evidence has increased the high or critical probability for the top nodes except for Safety Incidents. The node Cost of Disruption has the highest impact with High probability increasing to 70%. Among other nodes Average Delay Time and Average Number of Trains were also affected by the new evidence and their high state probabilities increased significantly. Fig 8 shows the scenario analysis results for the complete model.

**Fig 9:**
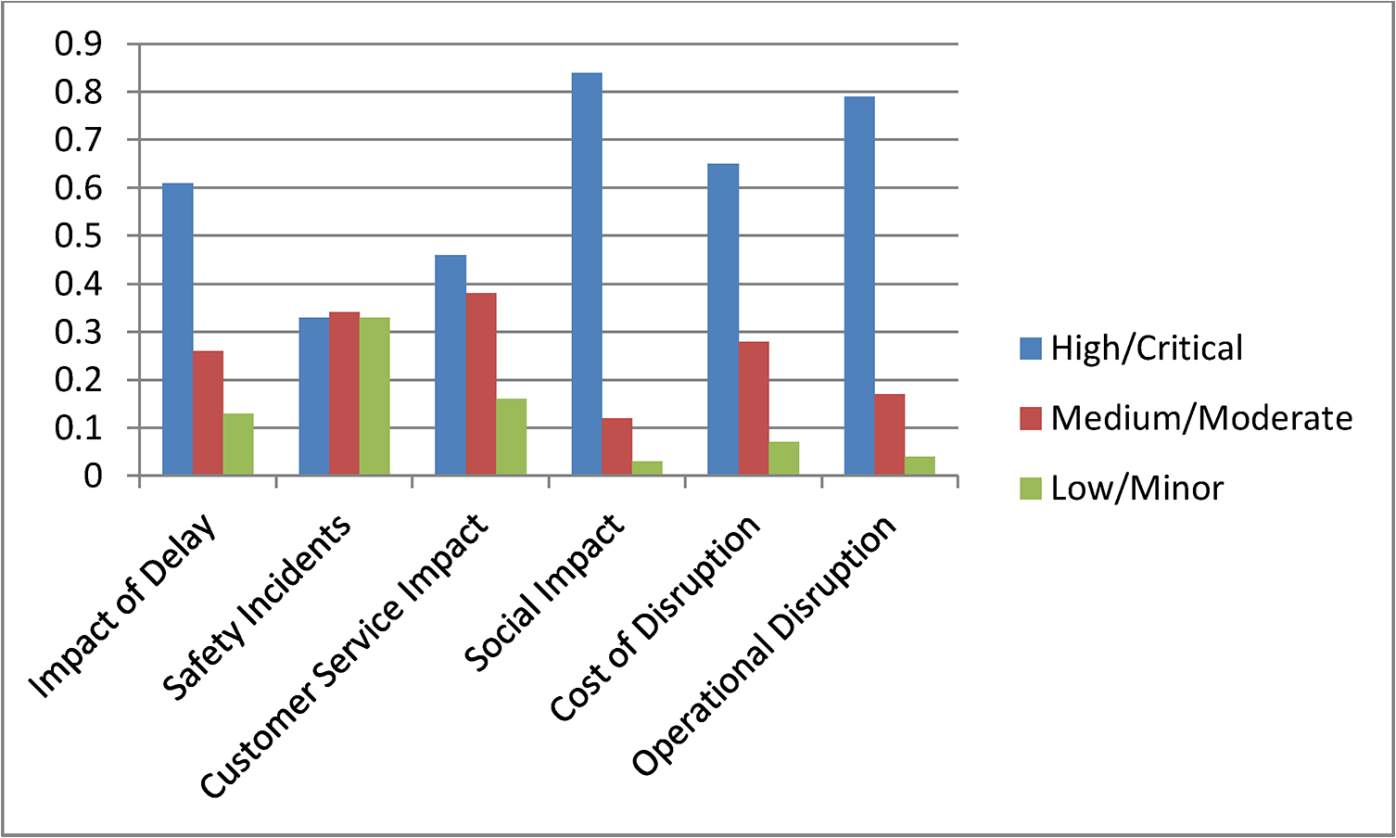
Probabilities for top level nodes for Scenario 2.

**Fig 10:**
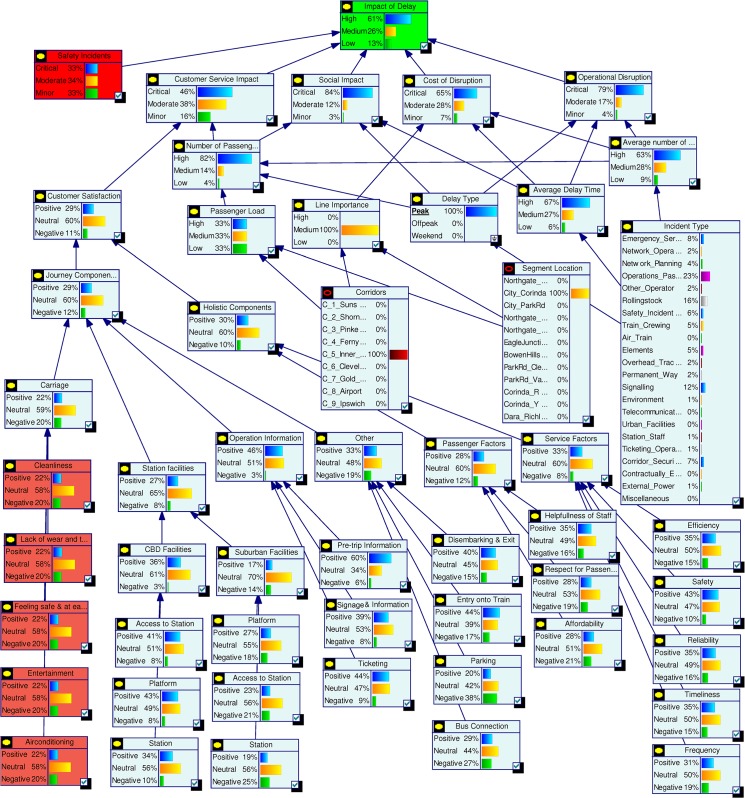
Analysis results for Scenario 2.

Scenario 2: **(**Assessing time criticality) Assume that a delay has occurred in the network and the time of the day is a weekday in the morning, i.e., a peak time. We set this evidence into the network as Peak = 100% for the node Delay type. The changes for the top two level nodes are shown in Fig 9, and Fig 10 shows the results for the complete model. We observe dramatic rises in the probabilities of High or Critical states of the nodes Impact of Delay, Customer Service Impact, Social Impact and Operational Disruption. Further analyses were conducted by setting the Delay type to Off-peak and Weekend states. These analyses further highlighted the significance of having fewer interruptions on the Impact of Delay during peak hours.

**Fig 11:**
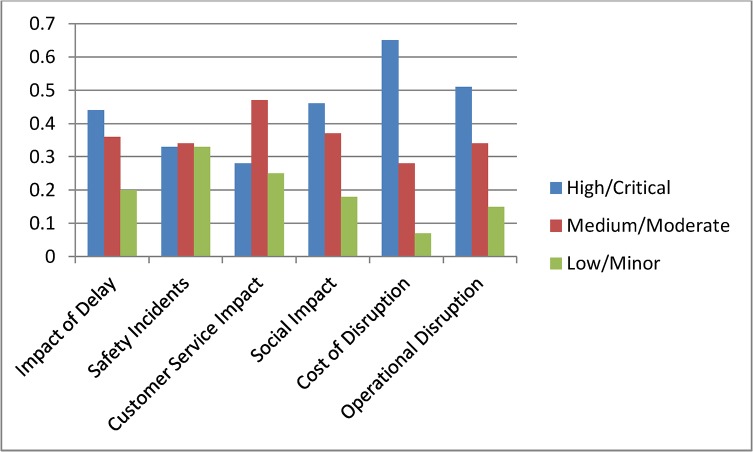
Probabilities for top level nodes for Scenario 3.

**Fig 12:**
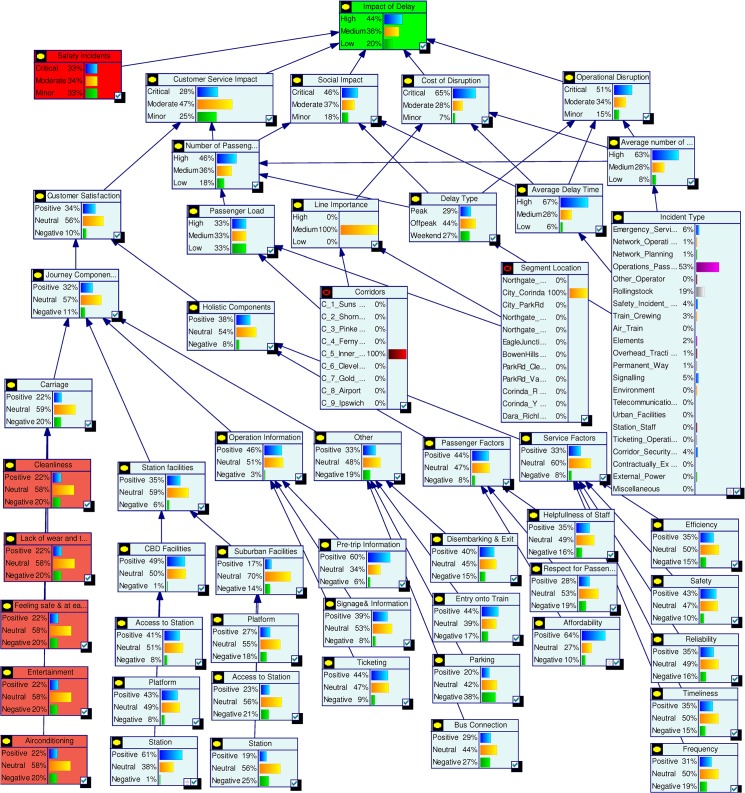
Analysis results for Scenario 3.

Scenario 3: **(**Assessing a policy change) Assume that Queensland Rail has recently upgraded its signalling system and expects signal related disruptions to reduce to about 5%. They have also introduced measures to improve customer satisfaction. Major upgrades were undertaken to improve city stations and experts at Queensland Rail believe that this will improve positive customer feedback for city stations significantly. Also, a new fare discount policy is introduced which will boost customer satisfaction in terms of affordability. We set this scenario into the model by setting the nodes Station (CBD), Affordability and Incident Type as Positive = 61%, Positive = 64% and Signalling = 5% respectively. Analysis results for top two level nodes are shown in Fig 11 and complete results are shown in Fig 12. The analysis shows significant improvement in Customer Satisfaction but only a minor effect on the Impact of Delay. The signal improvement does not have much effect on the delay impact.

**Fig 13:**
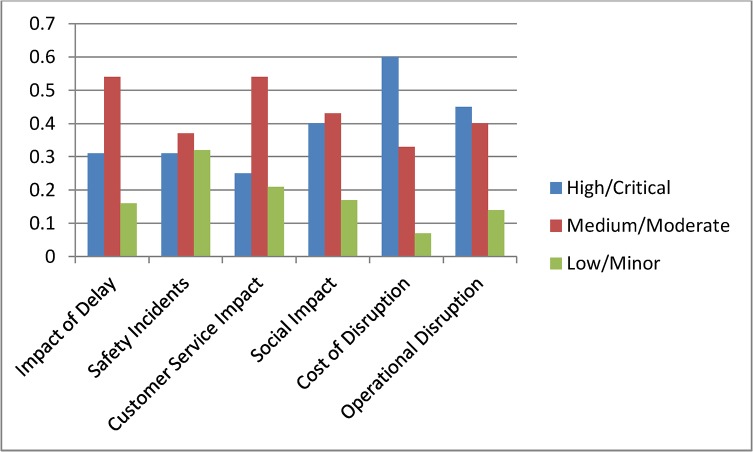
Probabilities for top level nodes for Scenario 4.

**Fig 14:**
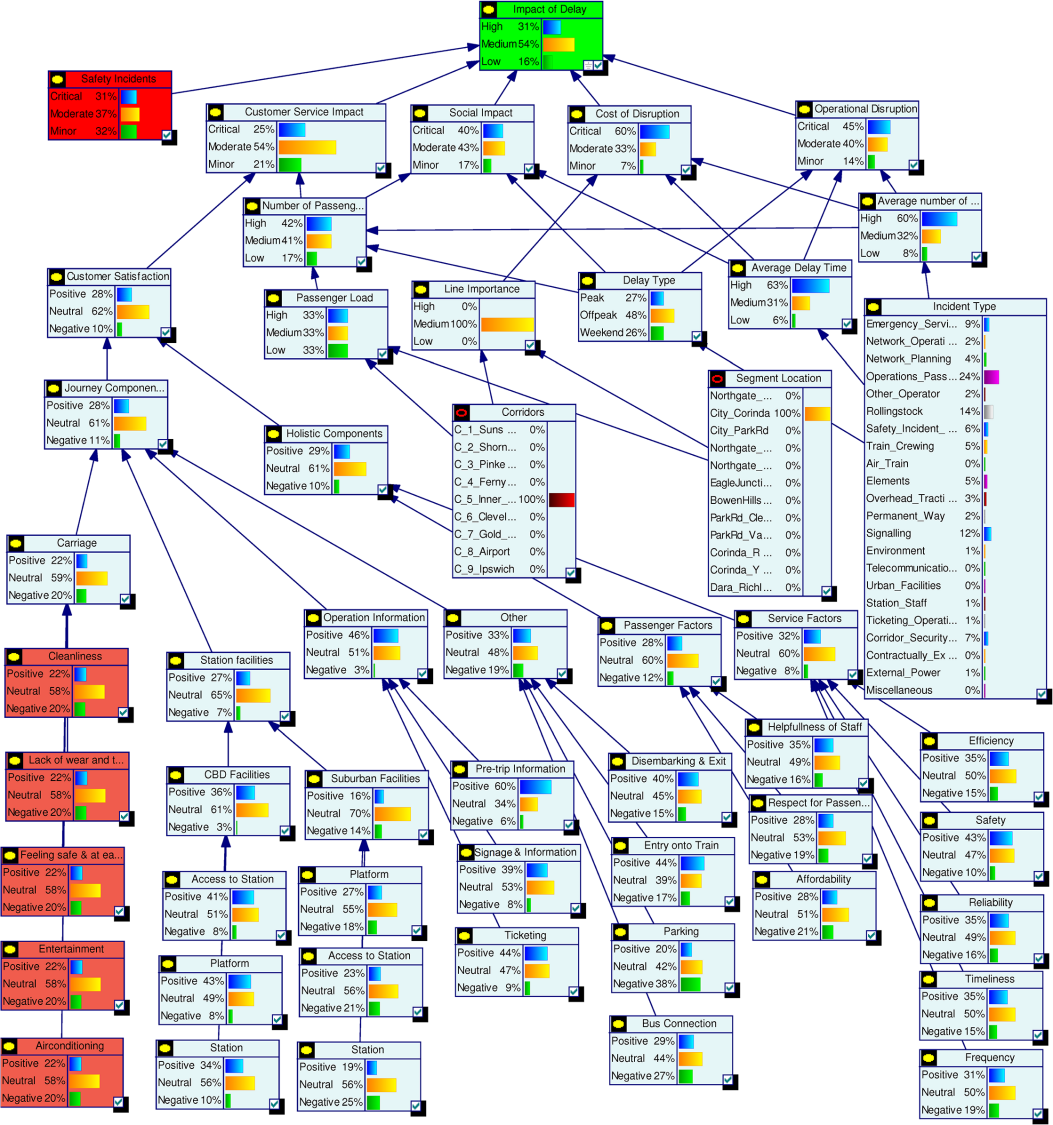
Analysis results for Scenario 4.

Scenario 4: **(**Assessing a target) Assume that Queensland Rail management has undertaken a strategic decision to reduce the High Impact of Delay probability from 45% to 30% and would like to identify the areas of improvement needed to do this. In this scenario our aim is not to assess the effect of making changes, but instead to determine what changes must be made to achieve a desired effect. We set the top node Impact of Delay to High = 31%. The analysis results are shown in Fig 13 but in this inverted scenario it is actually the changed nodes seen in Fig 14 that tell us what areas of investment to focus on to achieve the desired changes. We observe that in order to achieve the desired target objective the management needs to improve the performances of Cost of Disruption, Operational Disruption and Customer Service Impact significantly. Required changes for lower level nodes can be observed in Fig 14.

**Fig 15:**
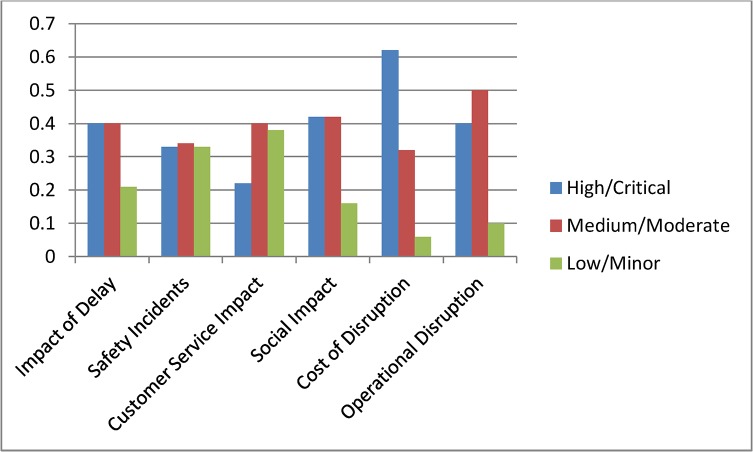
Probabilities for top level nodes for Scenario 5.

**Fig 16:**
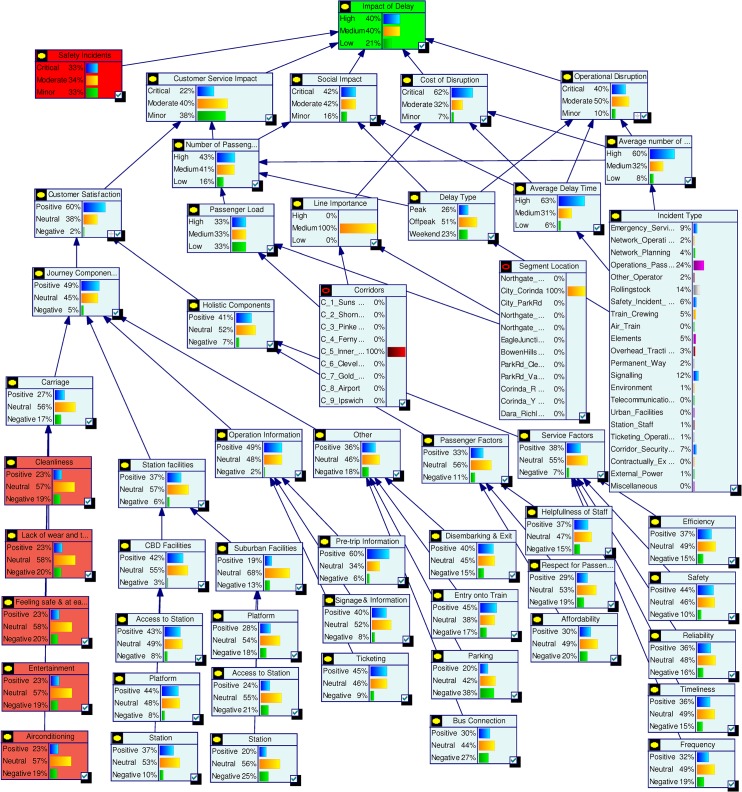
Analysis results for Scenario 5.

Scenario 5: **(**Assessing an action plan) Assume that management has decided to improve Queensland Rail’s “customer satisfaction” and “operational efficiency” Key Performance Indicators. The target is to double the positive customer satisfaction and reduce operational disruption by at least 10%. To analyse this scenario we set the Customer Satisfaction and Operational Disruption nodes as Positive = 60% and Positive = 40% respectively. From this we observed significant improvements in the Impact of Delay. Probabilities for corresponding parent nodes were also influenced in ways which highlight the level of improvements needed to achieve this action plan. Fig 15 shows top level results and Fig 16 shows the results for the complete model. Similar plans can be analysed all the way to the bottom node level.

#### Validation

In order to validate the model’s structure, we conducted a series of interviews with Queensland Rail experts in their Customer Service department. Confirmation was obtained that the factors (nodes) and their relationships represent the functional structure in the Queensland Rail Customer Service department. The Bayesian Network’s structure closely matched the structure of Queensland Rail’s survey questionnaire. Experience with the questionnaire by Queensland Rail experts further validates the appropriateness of the developed BN model.

The conditional probability tables represent the quantified model. CPTs were developed based on actual survey data, historical data, planning data and expert estimations currently used at Queensland Rail for performance evaluations. Expert confirmation was also obtained regarding the conformity of the model data with current practices at Queensland Rail.

The results of the BN analyses were validated using expert opinion. Experts with many years of experience in Queensland Rail’s Customer Service department confirmed that the Sensitivity Analysis and Influence Analysis results were as they expected. The Scenario Analysis results were also confirmed to be within the expert’s expected ranges.

#### Information communication

We communicated the results of this study to Queensland Rail as a Bayesian Network model developed in GeNIe [79]. The tool showed interactive features of the model and gave Queensland Rail managers an opportunity to use the models in practice. A detailed report was also provided explaining the model’s development process along with various analysis results. Throughout the various model development stages information was freely exchanged between Queensland Rail stakeholders and the researchers.

## Conclusion

In this article we have presented an approach that employs Bayesian Networks to integrate diverse data types from multiple sources, in order to better describe and understand complex enterprises and their KPIs. As an example of this approach, we presented the development of a complex system model for delay analysis at Queensland Rail. The model shows the interactions and dependencies between various factors influencing delays. Diverse data types were used to define the probabilistic characteristics of the model. The model can analyse various real life scenarios and identify key factors.

The strengths of the resulting model lie in its comprehensive representation of the actual system in a flexible manner. The model allows better analytical capabilities compared to other decision models such as CBA or MCDM through a transparent process ability to capture and assess variations in a complex system. The model can handle a wide variety of realistic scenarios. The analysis results from this model can be used, along with business goals, to make justified decisions in resource allocation and asset management. Although the current model was developed specifically for delay analysis at Queensland Rail, the approach can be applied easily to similar issues in other transport systems.

The study is a step forward to developing a comprehensive decision model capable of assisting complex decision making in large scale enterprises from disparate data. Future developments of this model will include devising decision models for operational decision support such as finding optimal maintenance schedule for large scale transport infrastructures.
